# Establishment and application of a rapid visual detection method for *Listeria monocytogenes* based on polymerase spiral reaction (PSR)

**DOI:** 10.1080/21655979.2022.2044262

**Published:** 2022-03-17

**Authors:** Moutong Chen, Tengyi Huang, Min Du, Xiaoxi Bai, Thanapop Soteyome, Lei Yuan, Caiying Bai, Haifeng Lan, Wei Hong, Fang Peng, Xin Fu, Gongyong Peng, Liyan Liu, Birthe V. Kjellerup, Zhenbo Xu

**Affiliations:** a Guangdong Provincial Key Laboratory of Microbial Safety and Health, State Key Laboratory of Applied Microbiology Southern China, Institute of Microbiology, Guangdong Academy of Sciences, Guangzhou, Guangdong, China; b Department of Laboratory Medicine, The Second Affiliated Hospital of Shantou University Medical College, Shantou, Guangdong, China; c Gmu-gibh Joint School of Life Sciences, Guangzhou Medical University, Guangzhou, Guangdong, China; d School of Food Science and Engineering, Guangdong Province Key Laboratory for Green Processing of Natural Products and Product Safety, Engineering Research Center of Starch and Vegetable Protein Processing Ministry of Education, South China University of Technology, Guangzhou, Guangdong, China; e Home Economics Technology, Rajamangala University of Technology Phra Nakhon, Bangkok, Thailand; f College of Food Science and Engineering, Yangzhou University, Yangzhou, Jiangsu, China; g Guangdong Women and Children Hospital, Guangzhou, Guangdong, China; h Department of Orthopaedic Surgery, The Third Affiliated Hospital of Guangzhou Medical University, Guangzhou, Guangdong, China; i Department of Critical Care Medicine, The Third Affiliated Hospital of Guangzhou Medical University, Guangzhou, Guangdong, China; j State Key Laboratory of Respiratory Diseases, National Clinical Research Center for Respiratory Diseases, National Center for Respiratory Medicine, Guangzhou Institute of Respiratory Health, the First Affiliated Hospital of Guangzhou Medical University, Guangzhou, Guangdong, China; k Department of Civil and Environmental Engineering, University of Maryland, College Park, MD, USA; l National Institute of Fundamental Studies, Kandy, Sri Lanka

**Keywords:** polymerase spiral reaction (PSR), rapid detection, *Listeria monocytogenes*, food-borne pathogen, *hlyA gene*

## Abstract

*Listeria monocytogenes* is a common foodborne pathogen that presents in various food products, posing important threat to public health. The aim of this study was to establish a rapid and sensitive method with visualization to detect *L. monocytogenes* based on polymerase spiral reaction (PSR). Primers targeting conserved *hlyA* gene sequence of *L. monocytogenes* were designed based on bioinformatics analyses on the current available *L. monocytogenes* genomes. The isothermal amplification PSR can be completed under constant temperature (65ᵒC) within 60 min with high specificity and sensitivity. Twenty-five reference strains were used to evaluate the specificity of the developed reaction. The results showed that the sensitive of the reaction for *L. monocytogenes* in purified genomic DNA and artificially contaminated food samples were 41 pg/μL and 10^3^ CFU/mL, respectively. It was 100-fold more sensitive than conventional PCR. In conclusion, this novel PSR method is rapid, cost-efficient, timesaving, and applicable on artificially contaminated food samples, providing broad prospects into the detection of foodborne microbes with the promising on-site inspection.

## Graphical abstract

**Figure:**
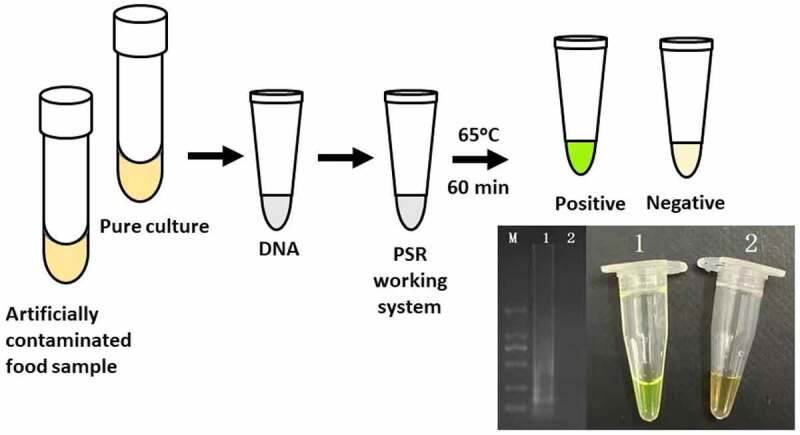

## Introduction

Food-borne disease caused by harmful microorganisms is the one of most important food safety issues effecting people’s life. Thousands of incidents of food poisoning occur each year caused by the food-borne pathogens [1]. According to the World Health Organization (WHO), more than 150 million food safety cases happen each year with 175,000 deaths in Southeast Asia which is higher than any other regions. Seventy-seven million people get illness caused by the contaminated food in America which come next to last [2]. Since the first isolation and description of *L. monocytogenes* in 1926, it has been shown the strong prevalence all over the world. Listeriosis is a food-borne disease among human and animals caused by *L. monocytogenes* with serious symptoms [3]. Foodborne listeriosis is important contributed by its severe clinical symptoms and high mortality rate of 30% among susceptible group, such as immunocompromised people, pregnant women, babies and the elders [4,5]. *L. monocytogenes* is able to present both in raw material and food processing.

With the increased attention to the food safety, the development of a rapid detection with visualization for identification of *L. monocytogenes* is significant and necessary.

Traditionally, the routine detection method of *L. monocytogenes* requires complicated operation and longtime involving the process of enrichment, colony formation and series of confirmation tests [6]. Except the conventional culture medium, there are detection methods based on the amplification of nucleic acid, including PCR and real-time PCR assays which developed well for the past decades [7]. However, *L. monocytogenes* detection and/or quantification in foods is still based on culture-dependent methods. Recently, researchers have raised much attention to novel isothermal amplification techniques due to the simple protocols rapid analysis and better performance compared with PCR [8,9]. Polymerase spiral reaction (PSR) is the one of novel isothermal amplification assays developed by Liu et al with efficient amplification of nucleic acid. The main primers Ft and Bt of PSR are composed of two segments, with a sequence between upstream and downstream is added to the front end of upstream primer F and downstream primer B of PCR, respectively. In the presence of Bst DNA polymerase and betaine, the upstream primers Ft and Bt bind to and extend the target sequence, respectively. After dissociation, each single chain contains the sequence we added and its complementary sequence, so it can be folded into a ring structure and continue to extend. The resulting new extension products can also be used as templates for reaction. Repeated primers combine, extend, unchain, single chain rotation and extended cycle will eventually form a series of complex structures with different molecular weight to achieve the purpose of nucleic acid amplification under isothermal conditions. This amplification can be completed under constant temperature with the advantages of simplicity, rapidity, high sensitivity and cost-efficient [10]. The expensive heating machine can be replaced by the simple water bath or other heating block. The whole amplification procedure can be completed within one hour by one pair of primer which span three distinct sequences of a target gene. The products of PSR can be measured based on the turbidity, electrophoresis of amplicons and DNA-specific fluorescent reaction in tubes, such as SYBR Green-I [11]. However, the application of PSR method on detection of *L.monocytogenes* especially in food samples has not been reported.

Therefore, the current study aimed to establish a rapid and cost-efficient assay to detect the *L. monocytogenes* by PSR technique based on *hlyA* gene which might have a contribution on solving food-borne outbreaks related to *L. monocytogenes*. Additionally, the limit of detection (LOD) and specificity were compared with the PCR assays. The applicability of the PSR in the food industry were evaluated by analysis of food samples.

## 2 Materials and methods

### 2.1 Bacteria strains

To standardize and evaluate the reaction system of PSR assay, a total of 25 bacteria strains were used in this study, including five standard strains and 20 non-target strains (Table 1). *L.monocytogenes* ATCC19116, ATCC19114, ATCC19115, ATCC15313, ATCC19113 were used as positive control. All strains used in this study had been preliminarily identified in the Lab of Clinical Microbiology, Zhongshan Supervision Testing Institute of Quality & Metrology. The strains were grown overnight in trypticase soy broth (TSB) at 37ᵒC with shaking at 200 rpm upon further use.

**Table 1.** Bacterial strains used in this study

		PCR	PSR
Reference strain no	No. of strains	hylA	hylA
Listeria monocytogenes ATCC19113	1	+	+
Listeria monocytogenes ATCC19114	1	+	+
Listeria monocytogenes ATCC19115	1	+	+
Listeria monocytogenes ATCC19116	1	+	+
Listeria monocytogenes ATCC15313	1	+	+
*Escherichia coli* O157:H7 ATCC43894	1	−	−
*Escherichia coli* O157:H7 ATCC43895	1	−	−
*Escherichia coli* O157:H7 E019	1	−	−
*Escherichia coli* O157:H7 E020	1	−	−
*Escherichia coli* O157:H7 E043	1	−	−
*Escherichia coli* O157:H7 E044	1	−	−
*Salmonella enterica* ATCC29629	1	−	−
*Salmonella enterica* ATCC14028	1	−	−
*Salmonella enterica* ATCC19585	1	−	−
*Salmonella enterica* ATCC13076	1	−	−
*Vibrio parahaemolyticus* ATCC27969	1	−	−
*Vibrio parahaemolyticus* ATCC17802	1	−	−
*Pseudomonas aeruginosa* ATCC27853	1	−	−
*Pseudomonas aeruginosa* C9	1	−	−
*Pseudomonas aeruginosa* C40	1	−	−
*Staphylococcus aureus* ATCC23235	1	−	−
*Staphylococcus aureus* ATCC25923	1	−	−
*Staphylococcus aureus* 10085	1	−	−
*Staphylococcus aureus* 10071	1	−	−
*Lactobacillus casei*	1	−	−

### 2.2 DNA extraction

Pure strains of all bacteria were used for preparation of genomic DNA after incubation in TSB(Huankai Microbial, China) overnight. All genomic DNA were isolated by DNA extraction Kit (Dongsheng Biotech, Guangzhou) according to the manufacturer’s instructions with quantity and quality measured using a Nano Drop 2000 (Thermo Fisher Scientific Inc, Waltham, MA, USA). The isolated DNA was stored at −20ᵒC for further usage.

### 2.3 Primer design

All current available *L. monocytogenes* genomes in GenBank database from NCBI were collected and comparative genomic analyses were performed for primer selection. *hlyA* gene is used in this study as detecting target. Only a single copy of this gene is present in the genome of pathogenic *L. monocytogenes*. This gene encoding listeriolysin O toxin which is necessary for virulence and is thus used for identifying *L. monocytogenes* in the presence of other Listeria strains. The primers were designed to identify the specific target *hlyA* of *L. monocytogenes* to detect the target and non-target microbes. The protocol of primer design was described as follows. The spiral primers (Ft and Bt) targeting the *hlyA* gene includea forward and reverse primer [10]. Primers used in this study were listed in Table 2, which were designed using Primer Premier 5.

**Table 2.** Primers sequence used for PSR

Target gene	Primers	Sequence (5’-3’)
hylA	Ft	ACACCAGGAGTTCCCATTGCAACCTCGGAGACTTA
	Bt	TTACCCTTGAGGACCACAGTAGCCTCCAGAGTGAT

### 2.4 Establishment of PSR assays

Twenty-five reference stains were used to develop and evaluate the specificity and sensitivity of PSR methods. The process of pure culture and DNA extraction were performed as described previously. The final volume of reaction system was 26 μL. The working system includes 20.0 mM Tris-HCl, 10.0 mM, (NH_4_)_2_SO_4_, 10 mM KCl, 8.0 mM, MgSO_4_, 0.1%, Tween 20, 0.7 M betaine (Sigma, USA), 1.4 mM of dNTP mix, 8 U of *Bst* DNA polymerase large fragment (NEB, USA), 1.6 μM (each) of the primers Ft and Bt, the specified amounts of genomic DNA and 1 μL mixture chromogenic agent. The total volume of reaction system was made up to 26 μL with nuclease free water. The reaction mixtures of PSR were heated at 65ᵒC for 60 min and terminated at 80ᵒC for 2 min. Nuclease free water instead of DNA template was used as blank control. The amplified products were analyzed by electrophoresis on 1.5% agarose gels.

Ratio of calcein and Mn^2+^, dosage of betaine and reaction time were compared to optimize the PSR condition. To investigate the effect of dye on the reaction, the ratio of calcein and Mn^2+^ from 1:20 to 1:2 were tested respectively. Simultaneously, betaine plays a significant role in DNA melting, it is necessary to confirm its concentration in PSR reaction. Moreover, to investigate the minimum reaction time required in a PSR run, 12 different time points had been studied, including 5 min, 10 min, 15 min, 20 min, 25 min, 30 min, 35 min, 40 min, 45 min, 50 min, 55 min and 60 min, respectively.

### 2.5 Specificity and sensitivity of PSR assay

The specificity of PSR assay was evaluated by the amplification of the genomic DNA extracted from five standard *L. monocytogenes* and 20 non-*L. monocytogenes* strains as listed in Table 1. Non-target bacteria include *Escherichia coli, Salmonella, Staphylococcus aureus, Vibrio parahaemolyticus, Lactobacillus casei* and *Pseudomonas aeruginosa*. Nuclease free water was added as blank control. The reaction of PSR was conducted under the corresponding conditions mentioned above.

To evaluate the sensitivity of the PSR assay, the genomic DNA of *L. monocytogenes* were serially 10-fold diluted by nuclease free water. The products of PSR were detected by 1.5% agarose gel electrophoresis. All the tests were performed in triplicate.

### 2.6 Application of PSR assay in rice products

The Application of PSR assay of detection for *L. monocytogenes* were conducted in rice products (Cantonese rice cake, steamed bread and rice noodle from Guangzhou Restaurant, Guangzhou). Twenty-five gram of frozen pastry (Guangzhou Restaurant, Guangzhou) was added to 225 mL of 0.9% NaCl which were sterilized as food samples and contaminated by *L. monocytogenes* strains. The concentrations of *L. monocytogenes* ATCC19113 DNA, extracted from serially diluted contaminated food samples, ranging from 10^8^–10 CFU/mL were subjected to PSR and PCR methods in triplicate [11].

## 3 Results

### 3.1 Establishment of PSR method for L. monocytogenes

**Figure 1.**
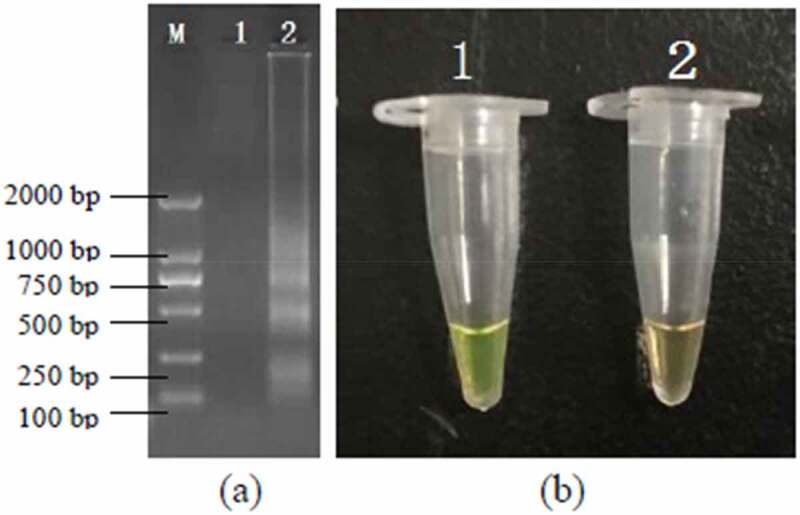
Results of the PSR assays for detection *invA* gene; (a) observation of amplification products with 1.5% agarose gel electrophoresis under UV light and fluorescence dye by naked eye (b); M, DNA marker; lane 1, negative control; lane 2, positive products (a); tube 1, positive products; tube 2, negative control.

The PSR assays for *hlyA* were established using *L. monocytogenes* ATCC19113. The amplified products were detected by 1.5% agarose gel electrophoresis, and the bands were observed under UV light (Figure 1(a)). Agarose gel analysis revealing the typical electrophoresis pattern of PSR amplified product, which is not a single band but a ladder pattern because the PSR method forms amplified products of various sizes consisting of alternately inverted repeats of the target sequence on the same strand. In addition, 1 μL mixture chromogenic agent (MgCl_2_ and calcein) we added into the PSR reaction system, wherein the dye color simultaneously changed from original to green in the positive sample, or water retained the original orange color (Figure 1(b)).

### 3.2 Specificity and sensitivity of PSR assay and its application

**Figure 2.**
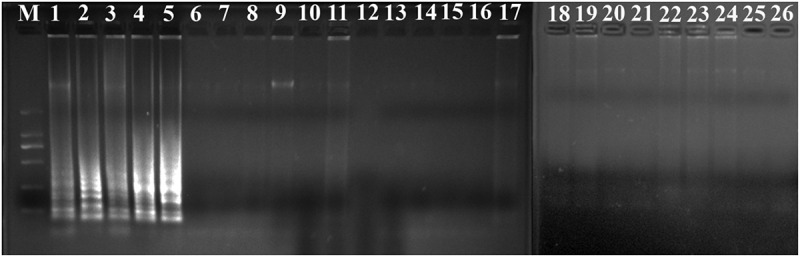
Specificity of PSR assay for detection *L. monocytogenes* strains with *hylA* genes by 1.5% agarose gel electrophoresis; M-DNA marker; lane 1–5, *L. monocytogenes* ATCC19116, ATCC19114, ATCC19115, ATCC15313, ATCC19113; lane/tube 6–25, non-*L. monocytogenes* strains of *Escherichia coli* O157:H7 (ATCC43894, ATCC43895, E019, E020, E043, E044), *Salmonella enteric* (ATCC29629, ATCC14028, ATCC19585, ATCC13076), *Vibrio parahaemolyticus* (ATCC27969, ATCC17802), *Pseudomonas aeruginosa* (ATCC27853, C9, C40), *Staphylococcus aureus* (ATCC23235, ATCC25923, 10085, 10071), and *Lactobacillus casei*; lane 26, negative control.

**Figure 3.**
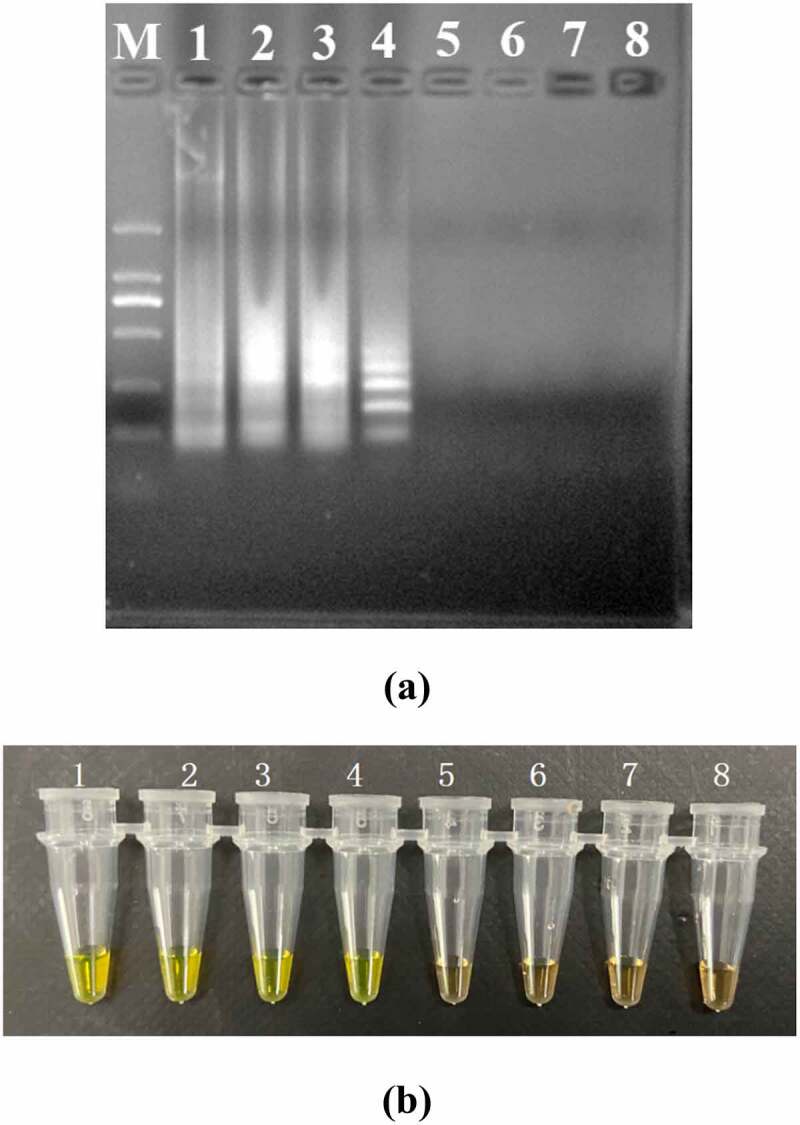
Sensitivity of the PSR assay in genomic DNA of *L. monocytogenes* with *hylA* genes by 1.5% agarose gel electrophoresis (a) and fluorescence dye by naked eye (b); M-DNA marker; lane/tube 1–8, 41 ng/μL, 4.1 ng/μL, 410 pg/μL, 41 pg/μL, 4.1 pg/μL, 410 fg/μL, 4.1 fg/μL, Negative control.

The PSR assay was evaluated for its specificity using five *L. monocytogenes* strains and 20 non-*L. monocytogenes* reference strains as control. Of the 25 strains, only the DNA of *L. monocytogenes* strains were amplified successfully. No cross-reaction was found with all the related non-target strains indicating the high specificity of the designed PSR primers (Figure 2). The analytical sensitivity of the PSR assay was measured using 10-fold serial dilutions of genomic DNA of *L. monocytogenes* ATCC19113. After amplification reaction, the results revealed that the LOD of PSR reaction was 41 pg/μL for *hlyA* (Figure 3). While that of the PCR was 4.1 ng/μL, indicating that the sensitivity of PSR assay was significantly higher than the traditional PCR method.

### 3.3 Application of detect L. monocytogenes in rice products

**Figure 4.**
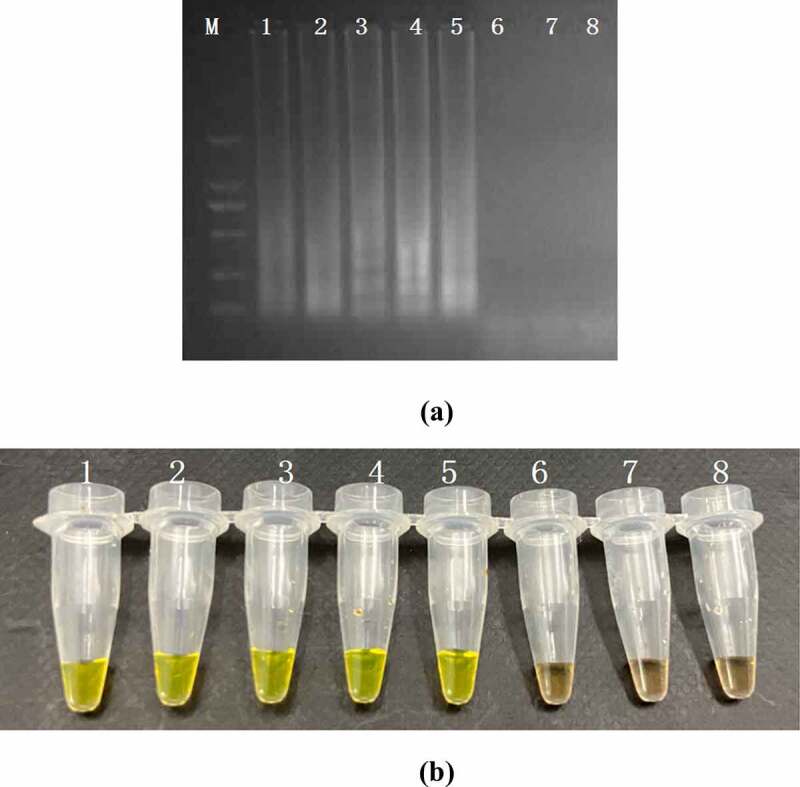
Sensitivity of the PSR assay in genomic DNA of *L. monocytogenes* with *hylA* genes from food samples: by 1.5% agarose gel electrophoresis (a) and fluorescence dye by naked eye (b); M-DNA marker; lane/tube 1–8, 10^7^ CFU/mL; 10^6^ CFU/mL; 10^5^ CFU/ mL; 10^4^ CFU/mL; 10^3^ CFU/mL; 10^2^ CFU/mL; 10^1^ CFU/mL, Negative control.

The sensitivity of PSR and PCR assay were performed by a series 10-fold diluted concentration (10^8^–10 CFU/mL) of DNA templates which were extracted from the artificially contaminated rice products. The results were subject to detect by electrophoresis in 1.5% agarose gels and fluorescence dye by naked eye (Figure 4). The sensitivity of PSR assay for *hlyA* gene in food samples were 10^3^ CFU/mL and no false positive amplification was observed, indicating high specificity of the established PSR assays.

## 4 Discussion

*L. monocytogenes* is an important food-borne pathogen around the world due to their resistance to antibiotics and adverse conditions, such as low pH, temperature and high salt concentration. It is found in various food commonly, especially fish, meat, poultry and dairy products [12]. Thus, the development of a rapid and intuitive detection method for this food-borne microbe is essential from the perspective of public health.

Normally, the confirmation of amplification reaction would be performed by the electrophoresis which is easier to cause the false positive results due to the process of open reaction cover. However, with the improvement of fluorescence reaction, results can be identified by the color change of reaction tube by naked eye which saves much time for whole detection. In current study, the entire reaction can be achieved within 60 min without the requirement of sophisticated equipment. This innovative isothermal amplification, PSR, has been used to detect various microbes, including *E. coli, Salmonella, P. aeruginosa, Candida albicans* and *V. parahaemolyticus* [10,11,13–15]. The PSR technique developed in this study can be specific to detect *L. monocytogenes* strains both in pure culture and food samples with 100 times higher sensitivity than PCR.

## 5 Conclusion

In conclusion, we have demonstrated that PSR assays could be useful and powerful tools for the rapid detection of *L. monocytogenes* by targeting on *hlyA* gene. Undoubtedly, the simplicity, rapidity and sensitivity of PSR assay make it an ideal routine diagnostic tool for rapid diagnosis of food-borne pathogens in both commercial and clinical fields that will strongly support the cooperation of patients and clinicians in endemic areas. With such advantages, the established PSR assay is a potentially highly complementary method to cultural approaches for quick and accurate detection of *L. monocytogenes* in foodstuff tested as part of outbreak investigations and other surveillance projects.

## Highlights

- The isothermal amplification method PSR can be completed under constant temperature within 60 min with high specificity and sensitivity.
- The developed PSR assay is 100-fold more sensitive than conventional PCR in the detection of *L. monocytogenes*.
- The PSR assay is applicable in the detection of *L. monocytogenes* in artificially contaminated food samples.
